# Development and feasibility of a set of quality indicators relative to the timeliness and organisation of care for new breast cancer patients undergoing surgery

**DOI:** 10.1186/1472-6963-12-167

**Published:** 2012-06-21

**Authors:** Marie Ferrua, Mélanie Couralet, Gérard Nitenberg, Sandrine Morin, Daniel Serin, Etienne Minvielle

**Affiliations:** 1 INSERM-COMPAQ-HPST project, Institut de Cancérologie Gustave Roussy, 114 rue Edouard Vaillant, Villejuif, 94805, FRANCE; 2 Haute Autorité de Santé, 2 avenue du Stade de France, St Denis la Plaine Cedex, 93218, FRANCE; 3 Institut Sainte Catherine, 1750 Chemin du Lavarin, Avignon, 84000, FRANCE

**Keywords:** Breast cancer, Quality indicators, Quality of health care, Timeliness of care

## Abstract

**Background:**

Because breast cancer is a major public health issue, it is particularly important to measure the quality of the care provided to patients. Survival rates are affected by the timeliness of care, and waiting times constitute key quality criteria. The aim of this study was to develop and validate a set of quality indicators (QIs) relative to the timeliness and organisation of care in new patients with infiltrating, non-inflammatory and metastasis-free breast cancer undergoing surgery. The ultimate aim was to use these QIs to compare hospitals.

**Methods:**

The method of QI construction and testing was developed by COMPAQ-HPST. We first derived a set of 8 QIs from consensus guidelines with the aid of experts and professional associations and then tested their metrological properties in a panel of 60 volunteer hospitals. We assessed feasibility using a grid exploring 5 dimensions, discriminatory power using the Gini coefficient as a measure of dispersion, and inter-observer reliability using the Kappa coefficient.

**Results:**

Overall, 3728 records were included in the analyses. All 8 QIs showed acceptable feasibility (but one QI was subject to misinterpretation), fairly strong agreement between observers (Kappa = 0.66), and wide variations in implementation among hospitals (Gini coefficient < 0.45 except for QI 6 (patient information)). They are thus suitable for use to compare hospitals and measure quality improvement.

**Conclusions:**

Of the 8 QIs, 3 are ready for nationwide implementation (time to surgery, time to postoperative multidisciplinary team meeting (MDTM), conformity of MDTM). Four are suitable for use only in hospitals offering surgery with on-site postoperative treatment (waiting time to first appointment after surgery, patient information, time to first postoperative treatment, and traceability of information relating to prognosis). Currently, in the French healthcare system, a patient receives cancer care from different institutions whose databases cannot as yet be easily merged. Nationwide implementation of QIs covering the entire care pathway will thus be a challenge.

## Background

Breast cancer is a major public health issue. It has the highest incidence amongst cancers in women (52,000 new cases in 2010) and is the first cause of death in women aged 35–65 years in France (11,300 deaths in 2008) [1]. However, measuring quality of care delivered to breast cancer patients is a challenging issue. In 2004, the United States Federal Agency for Healthcare Research and Quality (AHRQ) highlighted the paucity and need to develop validated quality measures to assess the quality of breast cancer care [2]. This need for “reliable, validated quality measures […] to afford accountability, improvement, and research” was reiterated many times in the USA and in Europe.

Since then, European guidelines for quality assurance, produced under the auspices of the European Commission, have listed 39 performance indicators for screening and diagnosis [3]. A 2010 position paper from the European Society of breast cancer specialists (EUSOMA) has proposed 17 main quality indicators (QIs) covering diagnosis, staging, surgery and loco-regional treatment, systemic treatment, counselling, follow-up and rehabilitation [4]. In France, the development of QIs in breast cancer care was flagged as a high priority in 2007. The treatment plan for each cancer patient has now to be discussed in a multidisciplinary team meeting (MDTM) held according to the rules laid down by the French National Cancer Institute (*Institut National du Cancer - INCa*) and the French National Authority for Health (*Haute Autorité de Santé - HAS*) [5,6].

Many of the QIs developed in the wake of the 2004 AHRQ report have been quality of life and patient satisfaction indicators [2]. However, more recently, in view of the importance given to waiting times by patients and many health care organisations, emphasis has also been placed on QIs measuring the timeliness of care [7-11]. The EUSOMA position paper proposes time to obtain mammography results, time between mammography results and the first consultation or between the core biopsy and surgical excision [4]. The second French national Cancer Plan (2009–2013) has urged that more is learnt about waiting times in order to reduce inequalities in access to care that may arise from undue delay [12]. Deviations from guidelines on timeliness can adversely affect 5-year survival rates [13-15], and patients who receive their test results promptly are less prone to anxiety [16-19].

To respond to this enquiry from the French health authorities, there is a need for simple, validated QIs that can be used to measure and compare quality of care in hospitals in order to identify room for improvement. Key methodological concerns, on which depends QI validity, are standardisation of data collection, reducing the workload of collection, and monitoring of QI inter-hospital variability. Only validated QIs can be implemented nationally or internationally, for instance in quality improvement programmes or paying for quality schemes, or used for public reporting.

The objective of this study was to establish the validity, for comparing hospitals, of a simple set of 8 easy-to-use QIs that assess the timeliness of key steps in the care of patients with infiltrating, non-inflammatory and metastasis-free breast cancer undergoing surgery.

## Methods

### Setting

The task of developing QIs for breast cancer management was delegated by the French authorities to the research project COMPAQH (*COordination for Measuring Performance and Assuring Quality in Hospitals*). COMPAQH’s remit is to develop QIs for monitoring quality in French hospitals and to design ranking methods and pay-for-quality programmes [20]. The project is run by INSERM (*French National Institute for Health and Medical Research*) and is sponsored by the French Ministry of Health and HAS which, together in 2003, listed 9 priority areas in need of quality improvement: pain management, practice guidelines, human resources management, iatrogenic events, nutritional disorders, access to care, taking account of patients’ views, coordination of care, and continuity of care. Quality of breast cancer care comes under the topic “practice guidelines” [21,22].

QI development in breast cancer care began in 2007 as a partnership between COMPAQH, *the French National Federation of Cancer Centres* (FNLCC), HAS, INCa, the *Société Française de sénologie et de pathologie mammaire (SFSPM)* and the *Collège National des Gynécologues et Obstétriciens français (CNGOF).* Attention was focussed on “new patients with infiltrating, non-inflammatory and metastasis-free breast cancer undergoing surgery within the institution” as this was the main concern of experts in the field and of consulting physicians. “New patients” were women with unilateral breast cancer who had never undergone treatment for breast disease, and who had not yet been seen in consultation or been admitted to the hospital for breast disease. We chose “infiltrating, non-inflammatory breast cancer” as this is the most common type of breast cancer (about 75 % of all cases) and constitutes a homogeneous population. Surgery is the primary treatment for most patients with metastasis-free T0 to T2 tumours.

### QI selection

**Figure 1:**
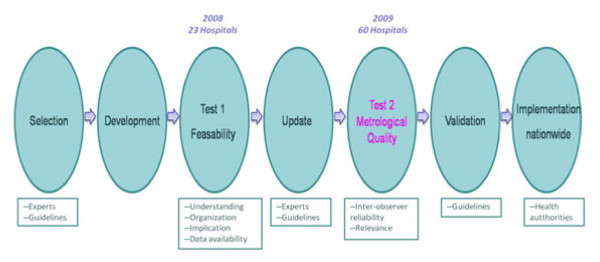
Steps in the development of QIs for breast cancer care by COMPAQ-HPST.

COMPAQ-HPST has a unique methodology for developing QIs [20,23,24] (Figure 1). The QIs for this study were designed with the help of experts in the field of breast cancer, submitted to SFSPM and CNGOF for their opinion, and selected by a working group of experts and consulting physicians in breast cancer. A list of criteria and items was drawn up for each QI. It was based on French practice guidelines for breast cancer management, legal regulations, and consensus-based guidance [21,22,25]. Factors influencing choice of criteria were: high level of evidence (level 1 whenever possible); discriminatory power (in view of variations observed in practice); practices in areas that were not subject to rapid change and where considerable improvement could be expected; applicability to all the hospitals in our sample; a precise working definition of the criterion that could be shared by all; and easy, standardised data collection. The number of criteria was restricted to 8 in order to lighten the burden of the participating hospitals. These QIs covered the care process as best possible.

### QI development and testing

Two tests were performed, first a preliminary test of QI feasibility in a small number of hospitals, then a larger scale test to measure QI performance.

For the preliminary test, performed in 2008, we asked 23 hospitals performing breast cancer surgery (including 20 comprehensive cancer centres) whether they would be willing to test the 8 QIs using their data for 2006. They assessed QI feasibility using a validated grid of 12 items exploring 5 dimensions (QI acceptability by staff, their understanding of the QI, their availability to respond in the allotted time, the ability of the hospital and the IT system to collect and handle the necessary data, and the workload) [26]. At the end of the test, we also assessed QI relevance as given by inter-hospital variability and deviation from expected performance.

For the second test (July–October 2009), we approached, via French hospital federations, all 633 hospitals performing breast cancer surgery in France (351 public, 231 private not-for-profit, and 51 private profit-making organisations). We assessed inter-hospital variability, internal validity (i.e. whether the QI really measured what it was intended to measure both from qualitative and quantitative points of view), and inter-observer reproducibility. Assessment was double-blind on 20 medical records from each of 14 hospitals. None of the QIs required adjustment.

### Data collection

For each test in each hospital, 80 patient records were analysed manually. This number was a compromise between acceptable workload and statistical validity [27,28]. The records were selected randomly from the PMSI database (*Programme de médicalisation des systèmes d’information*) which reports diagnosis-related group (DRG) statistics in a public hospital setting. The selected DRG code was breast cancer surgery. Apart from the 80 records for analysis, 20 additional records were also selected in the event of exclusions. Each hospital received an explanatory guide on the 8 QIs and a grid with instructions for its completion (available in French at http://www.compaqhpst.fr/data/indicateurs/12_GYC_V2_Grille_de_recueil_images.pdf).

### Statistics

For any given QI, at least 30 completed grids were required per hospital to support the assumption of a Gaussian distribution and to compute confidence intervals. This meant that, for any given QI, only those hospitals with at least 30 medical records for this QI were entered into the comparison among hospitals. Inter-hospital variability was given by QI score variance and the Gini coefficient which measures score dispersion. Variability (i.e. discriminatory power) is high if the Gini coefficient is under 0.2 and low if it is above 0.5 [29]. Internal validity was given by the overall concordance rate and inter-observer reproducibility by the Kappa coefficient [30,31]. We used SAS version 8.1 software (SAS Institute Inc, Cary, NC).

## Results

### Choice of QIs and feasibility test results

The working group established a list of 8 QIs: waiting time to first appointment with surgeon, time to surgery, time to postoperative MDTM, waiting time to appointment after surgery, time to first postoperative treatment, patient information, traceability of information relating to prognosis, and conformity of postoperative MTDM (Table 1).

**Table 1:** Tested QIs

QI 1	**Waiting time to first appointment with surgeon:** Median time to obtain first appointment with surgeon [1]
QI 2	**Time to surgery:** Proportion of patients undergoing surgery within 21 days of the first appointment with surgeon [1]
QI 3	**Time to postoperative MDTM:** Proportion of patients whose records were discussed in a MDTM held within 14 days of surgery [1]
QI 4	**Waiting time to appointment after surgery:** Proportion of patients given appointment relative to MDTM proposals within 14 days of MDTM [1]
QI 5	**Time to first postoperative treatment:** Proportion of patients whose first postoperative treatment was initiated within 30 days of urgery in the event of chemotherapy and within 56 days in the event of radiotherapy [9]
QI 6	**Patient information:** Proportion of patients receiving full information before surgery as detailed in article 40 of the French national Cancer Plan [15]
QI 7	**Traceability of information relating to prognosis:** Proportion of patients whose medical records provide all the diagnostic and prognostic information^a^ needed to initiate treatment [9,10,12]
QI 8	**Conformity of postoperative MDTM:** Proportion of patients for whom the postoperative MDTM was held according to the rules laid down in the French Health Ministry circular [1,13,16,23]

All the 23 hospitals (3 public, 20 cancer centres) taking part in the feasibility test completed and returned the grids. They randomly selected 2044 medical records: inclusion criteria were not met in 274 records, so 1770 (72 %) were included in our analysis. All QIs, except QI 1 (time to first appointment with surgeon), showed fair feasibility and high inter-hospital variability.

QI 1 presented ambiguities with regard to wording. Hospitals understood “time to first appointment” (QI 1) differently. Some hospitals thought that it was the date the patient’s medical record had been created, some that it was the date when the patient had called the hospital for an appointment, and some that it was the date on the GP’s letter requesting an appointment for the patient. Because of this ambiguity, QI 1 was reserved for the hospital’s information and was excluded from the hospital comparisons below.

### QI performance and inter-hospital comparisons

**Figure 2:**
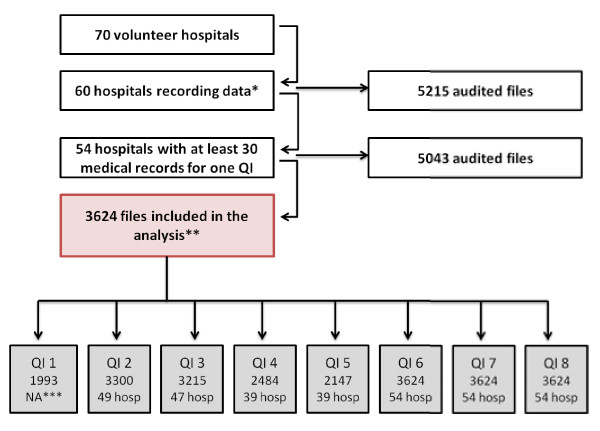
**Flow-chart of hospital and medical record numbers.** * Reasons for drop-outs: heavy workload (n = 5), unavailable data (n = 3), no response (n = 2). ** Reasons for exclusions: non-infiltrating or non-inflammatory breast cancer (mainly carcinoma *in situ*) (n = 414, 28 %), prior treatment for breast cancer (n = 597, 40 %), neo-adjuvant chemotherapy (n = 310, 21 %), bilateral breast cancer (n = 125, 8 %), and unavailable records (n = 41, 3 %). *** NA: not included in analysis as wording ambiguous according to results of feasibility test

Of the 633 hospitals performing breast cancer surgery, 70 accepted to participate in the testing of QI performance. Of the 70 participating hospitals, 60 completed and returned the grids (28 public, 10 private, and 22 not-for-profit privately owned organisations which included 20 cancer centres) (Figure 2). The reasons for the 10 drop-outs are given in the footnote to the flowchart in Figure 2. The 60 hospitals randomly selected 5215 medical records but we retained only those (n = 5043) that came from the 54 hospitals that had at least 30 records for at least one QI. From 6 to 21 hospitals, depending upon the QI, did not reach the required threshold of at least 30 medical records in order to be included in hospital comparisons.

The 54 hospitals were situated in different regions of France and differed in their status (public/private) and number of beds. All except one met the threshold volume of activity (> 30 breast cancer surgeries/year) that is required by French health authorities. The breakdown according to annual volume of activity was as follows: 28–80 operations (n = 6 hospitals), 122–197 (n = 11), 205–377 (n = 13), 476–945 (n = 18), 1036–1873 (n = 6).

We analysed 3624/5043 records (72 %) from the 54 hospitals. The main reasons for exclusions are given in the footnote to Figure 2. The incidence of missing data was by decreasing rank order: 40.6 % (1471/2153) for date of adjuvant therapy, 26.5 % (960/2664) for date of the postoperative appointment, 11.1 % (402/3222) for date of the postoperative MDTM, 8.8 % (319/3305) for the first appointment with the surgeon, and 0.4 % (9/3615) for the date of surgery.

QI 2 (“time to surgery”) was subject to misinterpretation: it was not clear whether it referred to (i) the appointment when the decision to perform surgery was taken or (ii) the appointment when the surgeon diagnosed suspected cancer and ordered tests before deciding to operate. Q2 was used, without modification, in the hospital comparisons.

**Figure 3:**
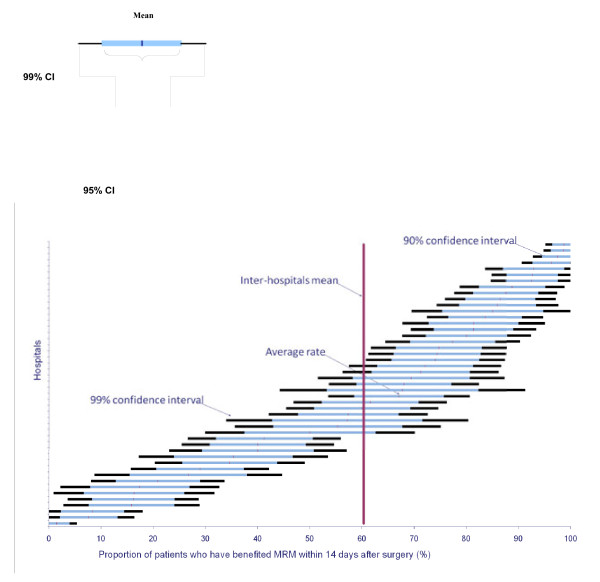
**Comparison among hospitals using QI 3.** (Proportion of patients undergoing postoperative MDTM 14 days after surgery). The result for each hospital (anonymous on the ordinate) is given by a horizontal line that represents confidence intervals (CI) (90 % CI - blue; 99 % CI - black). The vertical line gives the overall mean score for all hospitals and is used for benchmarking.

Table 2 gives for each QI the number of hospitals included in hospital comparisons, the mean score (i.e. mean percentage of patients with medical records that met the criteria outlined in Table 1) with ranges and standard deviations, and the Gini coefficient which measures dispersion. Scores varied widely, revealing substantial room for improvement in hospitals for all QIs, in particular QI 6 (patient information) and QI 8 (conformity of postoperative MTDM), which had the lowest mean scores. The Gini coefficient was <0.3 for all QIs (except QI 6 and QI 8) indicating that the power of the QIs to discriminate among hospitals was high (Figure 3). In the internal validity test, the overall discordance rate (lack of reproducibility) was 6.7 % (2.5–13.6). The Kappa coefficient score of 0.66 was indicative of fairly strong inter-observer reliability.

**Table 2:** QI conformity scores and discriminatory power

	**Hospitals (N)**	**Mean conformity score**^**a**^**% (range)**	**Standard Deviation**	**Discriminatory power (Gini coefficient)**
QI 1	Not applicable			
QI 2	49	58.2 (17.4–90.9)	21.5	0.19
QI 3	47	60.4 (1.4–98.7)	28.9	0.26
QI 4	39	84.5 (26.5–100)	15.9	0.08
QI 5	39	47.5 (11.2–91.5)	19.9	0.24
QI 6	54	12.8 (0–100)	25.3	0.73
QI 7	54	70.3 (4–98.7)	25.7	0.19
QI 8	54	46 (0–100)	44.7	0.44

^a^ Mean percentage of patients with medical records that met the criteria outlined in Table 1. There was significant divergence (<0.001) from expected performance for each QI.

## Discussion

Having defined quality as compliance with the care process, as this has been shown to be associated with patient outcomes, we developed 7 process QIs relating to the timeliness and organisation of breast cancer care. All 7 QIs were robust as indicated by their metrological properties and feasibility. In addition, all 7 highlighted considerable inter-hospital variability, thus revealing that there is substantial room for improvement in the quality of care.

Three of the 7 QIs are ready for nationwide implementation, namely, QI 2 (time to surgery), QI 3 (time to postoperative MDTM), and QI 8 (conformity of postoperative MDTM). Although some hospitals misunderstood the wording of QI 2 in the feasibility test, no change was made in the performance assessment test. The meaning has, however, since been clarified with a view to nationwide implementation of this QI. QI 2 now refers unambiguously to the date of the appointment when the decision to perform surgery is taken and not to the date of the appointment when the surgeon diagnoses suspected cancer and orders tests before deciding to operate.

The four other validated QIs (QI 6 – patient information, QI 4 – waiting time to first appointment after surgery, QI 5 – time to first postoperative treatment, and QI 7 – traceability of information relating to prognosis) are applicable only to hospitals that can offer both surgery and postoperative radio- or chemotherapy. Comparing all hospitals is rather hazardous as data for these QIs was often missing (11 %–40 % missing data). QI 6 had a very low mean conformity score (12.8 %) because of poor traceability of the information given to patients.

An 8^th^ QI we developed (QI 1 – waiting time to first appointment with the surgeon) proved to be too ambiguous to be used for comparisons among hospitals.

The external validity of our results may be considered satisfactory because (i) our patient sample was fairly representative as the 70 volunteer hospitals were a good reflection of available facilities for breast cancer care in France, (ii) it was homogeneous as we focussed on a subset of breast cancer patients, (iii) the number of audited and analysed medical records was large, (iv) results were insensitive to the reactive effects of testing and reactive settings because of the retrospective nature of the audit.

Our results reflect real-life conditions, i.e. the technical and organisational constraints observed when implementing QIs in hospitals. We anticipated the problems, taking into account the absence of validated quality measures of breast cancer care, leading to define quality as compliance with the process of care that has been shown to correlate with patient outcome. A systematic review, published in 2006, underlined the paucity of validated indicators of quality measures in breast cancer care and the need to develop “reliable, validated quality measures […] to afford accountability, improvement, and research” [32]. Several health care facilities have emphasized the importance of measuring timeliness of care from screening to pathology results, allowing to compare institutional performances, and (in these times of patient centred care) when patients were asked which aspects of care they would improve if they could, aspects relating to waiting times were most frequently mentioned [7]. So we decided to concentrate on timely access as a good representative of “quality care”.

Although we tried to forestall many of the problems that might arise when designing our QIs, we nevertheless had to contend with several hurdles.

The first hurdle was absence of all the required information in the French PMSI database which was used to randomly select the 80 medical records. We used restrictive inclusion criteria (“infiltrating, non-inflammatory breast cancer”) to obtain a homogeneous population. We excluded patients with carcinoma *in situ* and patients with prior breast cancer treatment. This had, however, to be done manually and represented a fairly heavy workload. The extra 20 records selected from the database to compensate for exclusions did not always make up for the recorded 28 % exclusion rate.

A second hurdle was that, in the French health care system, each hospital does not have access to all the data on a given patient. For example, QI 4 and QI 5 could not be calculated when follow-up or all care did not take place within the same hospital (e.g. appointment in private practice (QI 4), and appointment in one hospital with treatment in another hospital (QI 5)). The situation was even more complex when these hospitals had a different status (public, private, or not-for-profit).

A third hurdle, which also represent a limitation of our results, was that criteria on waiting times and delays are based on consensus among experts and not on standards derived from practice guidelines with a high level of evidence, which are normally used to construct QIs. Each country has its own standards [33]. The good practice guide produced in 2009 by the National Collaborating Centre for Cancer for NICE (National Institute for Health and Clinical Excellence) recommends not more than a 4-week delay from diagnosis to treatment, and starting chemotherapy or radiotherapy within 31 days of surgery [34]. In contrast, French guidelines published back in 2002 recommend a 21-day delay from the first appointment with the surgeon to surgery (similarly to the National Initiative on Cancer Care Quality (NICCQ) recommendation in the US [33]), a 30-day delay from surgery to chemotherapy, and 56 days from surgery to radiotherapy [25].

This hurdle could be partly overcome by using as targets the proportion of patients treated within set times. Such targets better satisfy health professionals for whom delays should reflect organisational constraints and not include patient-related causes (e.g. patient not turning up for the appointment, treatment postponed at the patient’s request). According to European guidelines, a threshold of 90 % is acceptable for ≤15 working days between the decision to operate and surgery and 70 % for ≤10 days [3]. According to EUSOMA, the minimum standard is >75 % and the target is >90 % for surgery performed within 6 weeks after the first diagnostic examination in the breast unit [4]. The Dutch auditing system has established a 90 % standard for 5 QIs [35]. However, a comparison with our results is difficult because of differences in QI definitions. Should the French health authorities take 90 % of patients registered in each time period as standard, there is much room for improvement in many hospitals as shown in Table 2.

Recent experiences in Europe and the USA have shown that QI implementation at a local [33]. or national level using a variety of methodologies can improve the quality of care of breast cancer patients but that this takes time [35-37]. According to the Dutch experience, none of 9 QIs met standards in 2002 whereas 4 did in 2008, with a significant improvement in all 9 QIs. Because hospitals simply perform better when they know they are being evaluated (Hawthorne effect), but also because comparison is able to promote a better registration process and compliance with best clinical practice, improvements can be expected in France also.

Whether QI scores may qualify hospitals in the certification of breast cancer centres is a moot point. We could propose to follow the example of the National Quality Measures for Breast Care (NQMBC), which uses the degree of participation to on-line registration of the answers to a set of quality questions to grant 3 levels of certification for quality breast health care [17,36].

## Conclusions

Our selected QIs on timeliness of breast cancer care proved feasible and applicable in the clinic. Their implementation was highly dependent on care organisation, patient behaviour, and the quality of the information systems used by French hospitals. Future QIs should cover the entire care pathway from before to after hospital consultations and admission, and should include the patient’s perspective [7,38]. The COMPAQ-HPST project is currently focusing on the construction of QIs covering care from an abnormal screening result right through to post-treatment follow-up, as in the ambitious programme developed by the American Society of Clinical Oncology (ASCO) [39]. This is a challenge because of the need to merge patient databases that are managed by hospitals using information systems that are often not compatible and of the need to guarantee access to these data. The challenge is even greater at the European level where account has to be taken of differences in the organisation of care among countries.

## Competing interests

The authors declare they have no competing interests.

## Authors’ contributions

MF participated in the acquisition of the data, was primarily involved in the conception, design, screening of all levels and drafting of the manuscript. MC assisted with data abstraction, and was responsible for the interpretation and statistical analysis of the data. GN has made substantial contribution to design of the manuscript, has been involved in the drafting and has given final approval of the version to be published. SM has been involved in the conception, design of the manuscript and in the acquisition of the data. DS revised the manuscript and brought important intellectual content. EM revised critically the manuscript, collaborated in conceptualizing the article elements and has given final approval of the version to be published. All authors read and approved the final manuscript.

## Pre-publication history

The pre-publication history for this paper can be accessed here:

http://www.biomedcentral.com/1472-6963/12/167/prepub
